# Macropinocytosis, mTORC1 and cellular growth control

**DOI:** 10.1007/s00018-017-2710-y

**Published:** 2017-11-08

**Authors:** Sei Yoshida, Regina Pacitto, Ken Inoki, Joel Swanson

**Affiliations:** 1 Department of Microbiology and Immunology, University of Michigan Medical School, Ann Arbor, MI 48109-5620 USA; 2 Department of Integrative and Molecular Physiology and Internal Medicine, Life Sciences Institute, University of Michigan, Ann Arbor, MI 48109 USA

**Keywords:** Macropinocytosis, mTORC1, Small GTPase, Phosphoinositide, Cancer

## Abstract

The growth and proliferation of metazoan cells are driven by cellular nutrient status and by extracellular growth factors. Growth factor receptors on cell surfaces initiate biochemical signals that increase anabolic metabolism and macropinocytosis, an actin-dependent endocytic process in which relatively large volumes of extracellular solutes and nutrients are internalized and delivered efficiently into lysosomes. Macropinocytosis is prominent in many kinds of cancer cells, and supports the growth of cells transformed by oncogenic K-Ras. Growth factor receptor signaling and the overall metabolic status of the cell are coordinated in the cytoplasm by the mechanistic target-of-rapamycin complex-1 (mTORC1), which positively regulates protein synthesis and negatively regulates molecular salvage pathways such as autophagy. mTORC1 is activated by two distinct Ras-related small GTPases, Rag and Rheb, which associate with lysosomal membranes inside the cell. Rag recruits mTORC1 to the lysosomal surface where Rheb directly binds to and activates mTORC1. Rag is activated by both lysosomal luminal and cytosolic amino acids; Rheb activation requires phosphoinositide 3-kinase, Akt, and the tuberous sclerosis complex-1/2. Signals for activation of Rag and Rheb converge at the lysosomal membrane, and several lines of evidence support the idea that growth factor-dependent endocytosis facilitates amino acid transfer into the lysosome leading to the activation of Rag. This review summarizes evidence that growth factor-stimulated macropinocytosis is essential for amino acid-dependent activation of mTORC1, and that increased solute accumulation by macropinocytosis in transformed cells supports unchecked cell growth.

## Introduction

Macropinocytosis is an endocytic process by which cells engulf relatively large volumes of extracellular fluid solutes, including nutrients, through movements of the plasma membrane [1, 2]. Subsequent organelle fusion reactions deliver internalized solutes into endolysosomal compartments, where macromolecules may be degraded by lysosomal hydrolases into constituent subunits for anabolic metabolism. Macropinocytosis was originally called pinocytosis [3, 4], but was later renamed to distinguish it from smaller endocytic vesicles such as clathrin-coated vesicles. Growth factors, cytokines, chemokines, pathogens, and the tumor promoter phorbol myristate acetate (PMA) can induce macropinocytosis. Macrophages and dendritic cells constitutively exhibit macropinocytosis, as do cells transformed by oncogenic mutations of K-Ras and v-Src [5, 6]. Aberrant activation of macropinocytosis has been implicated in cancer progression [7, 8], neurodegenerative diseases [9], atherosclerosis [10], and renal dysfunction [11].

Extracellular nutrients and growth factors can regulate cell growth, quiescence, and survival. In response to nutrient availability and growth factor stimulation, cells grow and proliferate by increasing anabolic metabolism. Mechanistic target of rapamycin (mTOR) is an evolutionarily conserved serine/threonine kinase that plays key roles in stimulating cellular anabolic processes and inhibiting catabolic processes such as autophagy in response to growth factors and nutrient availability. TOR was originally identified in yeast as a target protein of rapamycin, a macrolide compound that is now widely used in clinical settings as an immunosuppressant, anti-restenotic, and anti-cancer agent [12–15]. mTOR forms at least two distinct multi-protein complexes termed mTOR complex 1 (mTORC1) and mTORC2 [16–20]. Both complexes contain mTOR as a core kinase and the common subunits mLST8 (also known as GβL) [20] and DEPTOR [21]. mTORC1 [15] contains the specific subunits, raptor [18, 19] and PRAS40 [22–24], while mTORC2 contains rictor [17], mSIN1 [25, 26], and PROTOR [27]. While mTORC2 plays important roles in actin cytoskeleton reorganization, cell migration, survival, and glucose metabolism, mTORC1 has been shown to be essential in cell growth and a wide array of cellular metabolic processes [28–30]. In response to a variety of stimuli, including amino acids, glucose, growth factors, cytokines, and PMA [31–33], mTORC1 stimulates cell growth and proliferation by enhancing the rate of cellular protein synthesis, and lipid and pyrimidine/purine biogenesis [34]. Aberrant activation of mTORC1 plays key pathological roles in the development of diseases such as cancer, type 2 diabetes, atherosclerosis, and neurodegeneration [28, 29, 34–37]. Thus, the mechanism of mTORC1 activation and its roles in metabolic regulation have attracted intense interest in basic and clinical sciences.

Macropinocytosis and mTORC1 activation share many common mechanisms for their induction, and recent studies have demonstrated that macropinocytosis contributes to cell growth by stimulating mTORC1 activity [2, 7, 8, 38–42]. This review compares the molecular mechanisms underlying the induction of macropinocytosis and mTORC1 activity, and discusses crucial roles of macropinocytosis in the assimilation of nutrients for cell growth.

## mTORC1 activity is regulated by Rag and Rheb

**Fig. 1:**
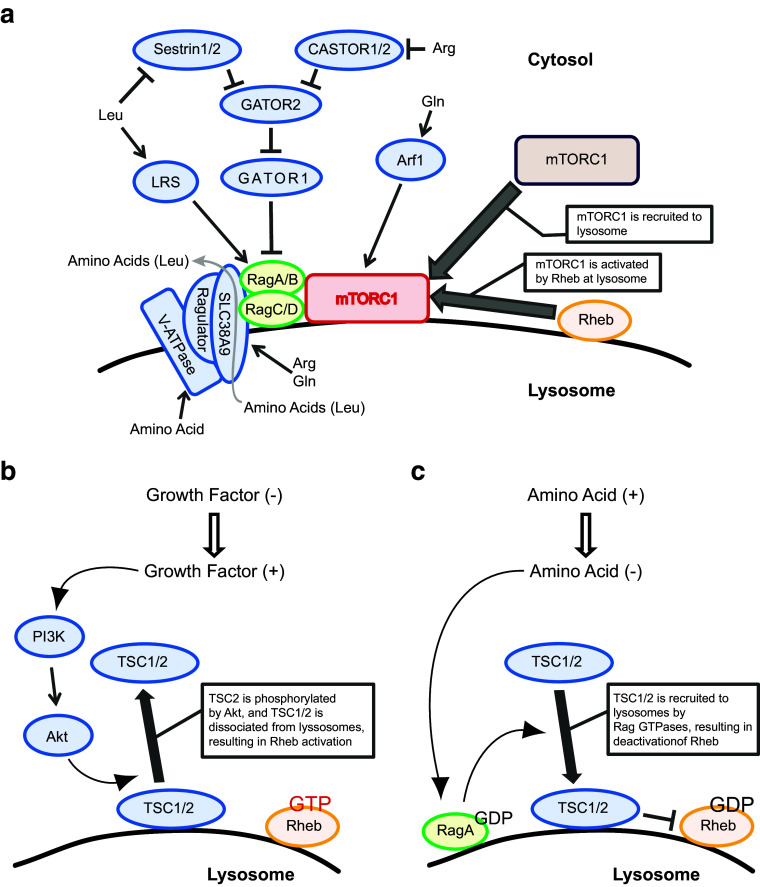
Amino acid- and growth factor-induced mTORC1 activation. **a** The mechanism of amino acid-induced mTORC1 activation. mTORC1 is recruited to lysosomes by amino acid stimulation. Through V-ATPase and SLC38A9 on lysosomal membranes, amino acids such as arginine (Arg) and glutamine (Gln) modulate the function of protein complex Ragulator, leading to Rag activation. Arg and Gln are detected by SLC38A9. Once Rag is activated, mTORC1 is recruited to lysosomes via the interaction between Rag and raptor, followed by mTORC1 activation by Rheb. Upon binding arginine, SLC38A9 transports amino acids, such as leucine (Leu), from the lysosomal lumen into cytoplasm. GATOR1 and GATOR2 regulate Rag function. Rag is inhibited by GATOR1, which is inhibited by GATOR2. Sestrin1/2 and CASTOR1/2 inhibit GATOR2, and detect Leu and Arg, respectively, in cytosol. The interaction of these amino acids with their target proteins results in the reversal of inhibition by GATOR2. Leucyl-tRNA synthetase (LRS) can also activate Rag and detect Leu in the cytosol. Gln in the cytosol is detected by an Arf1-dependent mechanism, followed by Rag activation. **b** The mechanism of growth factor-induced Rheb activation. Growth factor stimulation induces the PI3K–Akt pathway. Akt phosphorylates TSC2, which is located at lysosomal membrane as a protein complex with TSC1. After phosphorylation, the TSC1/2 complex dissociates from the lysosome. TSC1/2 is a Rheb GAP, so loss of TSC1/2 complex from the lysosomal membrane allows Rheb to be activated (Rheb-GTP). **c** The mechanism of amino acid-modulated Rheb deactivation. Depletion of amino acids from culture medium induces deactivation of RagA (GDP form). Inactivated RagA triggers TSC1/2 recruitment to lysosomes, resulting in deactivation of Rheb (Rheb-GDP)

The small GTPases Rag and Rheb coordinately stimulate the activity of mTORC1 on the surface of the lysosome [43–45] (Fig. 1a). Mammalian cells contain four isoforms of Rag, Rag A, B, C, and D, which form heterodimers comprised of RagA or B with RagC or D in a functional conformation, and which are activated by amino acids such as leucine and arginine. The Rag heterodimer interacts with a pentameric protein complex called Ragulator, which consists of the proteins p18 (LAMTOR1), p14 (LAMTOR2), MP1 (LAMTOR3), C7ORF59 (LAMTOR4), and HBXIP (LAMTOR5), and associates with the lysosomal membrane [44]. Ragulator functions as a scaffold for the Rag heterodimer to localize on the lysosomal membrane and to stimulate GTP-binding by RagA or RagB through its guanine nucleotide exchange factor (GEF) activity. Amino acids in the lysosomal lumen play a key role in triggering a conformational change of the transmembrane vacuolar H^+^-ATPase (v-ATPase), which activates the RagA/B GEF activity of Ragulator [46, 47]. In addition, SLC38A9, a lysosomal transmembrane protein, interacts with the v-ATPase and activates Ragulator by sensing luminal arginine [48–50]. Upon binding arginine, SLC38A9 transports leucine and other amino acids from the lysosomal lumen into cytoplasm [51]. Cytosolic arginine and leucine can activate the Rag heterodimer by inhibiting the inhibitory activity of a GTPase-activating protein (GAP) for RagA/B [52] (Fig. 1a). GATOR1, a trimeric protein complex consisting of DEPDC5, Nprl2, and Nprl3, is expressed on the lysosomal membrane and functions as a GAP for RagA/B. Furthermore, GATOR1 is inhibited by another pentametric protein complex, GATOR2 [53]. Thus, GATOR2 activates the Rag heterodimer by inactivating GATOR1. Sestrin1 and/or Sestrin 2 directly interact with and inhibit GATOR2, and suppress mTORC1 function [54, 55]. Sestrin bears a leucine-binding pocket in close proximity to its GATOR2 binding site, and the binding of leucine to Sestrin relieves its inhibitory effect on GATOR2. Thus, cytosolic leucine activates mTORC1 by inhibiting GATOR1 through its binding to Sestrin1/2. Similarly, cytosolic arginine activates mTORC1 by inhibiting GATOR1 through its binding to CASTOR1. CASTOR1 forms a homodimer or a heterodimer with CASTOR2 and inhibits GATOR2. Similar to the mode of Sestrins, arginine binding to CASTOR1 blocks its interaction with GATOR2 and relieves the CASTOR1 inhibitory effect on GATOR2, thereby activating RagA/B signaling [54–57]. Glutamine also stimulates mTORC1 [58]. However, it remains unclear whether glutamine itself functions as a signaling molecule for activating mTORC1. Rather, either glutamine stimulates the influx of leucine by acting as an efflux solute through a SLC7A5–SLC3A2 heterodimeric antiporter, or the glutamine metabolite α-ketoglutarate stimulates mTORC1 by activating the Rag heterodimer [59, 60]. It has also been reported that glutamine can activate mTORC1 in a manner dependent on Arf1 but not Rag small GTPase [58]. Thus, RagA/B-dependent activation of mTORC1 occurs by amino acids detected in the cytosol but reaching mTORC1 from within lysosomes or endolysosomes.

Activated Rag recruits mTORC1 to the lysosomal membrane through its interaction with Raptor [44, 61]. There, Rheb directly activates mTORC1 [15, 62, 63] (Fig. 1). Rheb itself is activated by signals from growth factor receptors [64] (Fig. 1b). Upon growth factor stimulation, active phosphoinositide 3-kinase (PI3K) synthesizes PIP_3_, which recruits PDK1 and Akt to the plasma membrane where Akt is phosphorylated and activated by PDK1 and mTORC2. Subsequently, active Akt on the lysosomal membrane phosphorylates and inhibits tuberous sclerosis complex 2 (TSC2), a GAP for Rheb in a larger complex comprised of TSC1, TSC2 and TBC1D7 [Tre2–Bub2–Cdc16 (TBC)1 domain family number 7] [65–67]. Alternatively, the RAS–MEK–ERK–RSK pathway phosphorylates and inactivates the TSC complex in response to growth factors, cytokines, and PMA [31, 32, 68–72]. The phosphorylation of TSC2 by Akt induces the dissociation of the TSC complex from the lysosomal membrane, consequently permitting GTP-loading of Rheb and subsequent mTORC1 activation [64, 72, 73]. The molecular mechanism by which Akt reaches the lysosome to phosphorylate TSC2, and how the phosphorylation of TSC2 leads to its dissociation from the lysosomal membrane are still unknown. Recent studies demonstrated that the dissociation of the TSC complex from lysosomes is also triggered by amino acid stimulation (Fig. 1c) [73, 74]. Under amino acid starvation conditions, the GDP-bound form of RagA (inactive) interacts with and recruits TSC2 to the lysosomal membrane. Conversely, GTP-bound RagA (active) is unable to retain the TSC complex on the lysosomal membrane. Thus, both growth factor-mediated TSC2 phosphorylation and amino acid-induced RagA activation induce the dissociation of the TSC complex and, consequently, stimulate Rheb-dependent mTORC1 activation. In addition to these mechanisms, a recent study demonstrated that arginine can directly inhibit the interaction between the TSC complex and Rheb, thereby supporting Rheb activation in response to amino acid availability [75].

## Involvement of endocytosis and autophagy in mTORC1 activation

Given that the cytosolic face of the lysosomal membrane serves as a platform for numerous proteins and protein complexes that mediate amino acid- and growth factor signaling for mTORC1 activation, it can be hypothesized that processes important for endosomal and lysosomal trafficking play key roles in the regulation of mTORC1 activity [76–78]. In addition to Rag and Rheb, other small GTPases associated with endocytosis contribute to the activation of mTORC1. In *Drosophila* S2 cells [79], mTORC1 activation was decreased by knockdown of Rab5 or Arf, which are important for endocytic membrane trafficking. Similarly, knockdown of mammalian Rab5 or Arf1 decreased mTORC1 activity in HEK293 or murine embryonic fibroblast (MEF) cells. Ectopic expression of dominant-active Rab5(Q79L) in HEK293 cells specifically blocked activation of mTORC1 by amino acids but not glucose, implicating Rab5-related endocytic traffic in amino acid-dependent mTORC1 activation [79]. Ectopic expression of active Rab5 often generates unusual vesicles containing both the early endosome marker EEA1 and the late endosome/lysosome marker LAMP1, indicating that aberrant Rab5 activation causes a defect in early-to-late endosome conversion [80]. Consistent with this observation, ablation of hVps39, which plays a role in the early-to-late endosome conversion, produced hybrid endosomes and inhibited insulin-induced mTORC1 activation [80]. mTORC1 localized to these hybrid endosomes, suggesting that the maturation or integrity of the late endosome/lysosome was critical for proper activation of mTORC1. It remains unclear whether Rheb localizes to these hybrid endosomes, and whether the dissociation of the TSC complex from these organelles occurs in response to growth factor stimulation. Together, these reports suggest that the transition from early to late endosome, regulated by Rab5, is required for mTORC1 activation.

As noted above, the GTPase Ras functions as an upstream suppressor of TSC2 via the ERK pathway [31, 71]. Expression of dominant active Ras(Q61L) in HEK293T cells induced TSC2 phosphorylation [71], and stimulated mTORC1, as indicated by S6K1 phosphorylation. Thus, Ras functions upstream of Rheb to stimulate mTORC1 activity. mTORC1 activation by Ras(Q61L) was blocked by amino acid starvation in fibroblasts [65], suggesting that Ras does not act downstream of amino acid sensing machineries to activate mTORC1. However, these observations leave open the possibility that active Ras acts upstream of amino acid sensing machineries to induce mTORC1 activation. In addition, recent studies demonstrated that ablation of the GTPase Rac1 attenuated growth factor-induced mTORC1 and mTORC2 activation in MEFs and HeLa cells [40, 81]. Immunofluorescence staining showed that Rac1 co-localized with mTORC1 and mTORC2 at the plasma membrane in response to serum stimulation [81]. As both Ras and Rac regulate endocytic pathways, these reports also suggest the involvement of endosomal traffic in mTORC1 activation. Interestingly, active Ras acts upstream of Rac1 to stimulate actin cytoskeleton reorganization, membrane ruffling, and macropinocytosis [1, 82].

Another activity in which mTORC1 is responsive to lysosome function is macroautophagy, a process in which cytoplasm is sequestered into membranous autophagosomes that, like macropinosomes, fuse with lysosomes to allow macromolecule hydrolysis and nutrient recycling. Inhibition of cellular mTORC1 activity stimulates autophagy [30], and amino acids recovered by autophagy can activate mTORC1 [51, 83, 84]. Thus, both heterophagy—the assimilation of exogenous nutrients by endocytic activities—and autophagy—the degradation of cytoplasmic contents—can provide amino acids for activation or reactivation of mTORC1.

## Mechanisms of macropinosome formation

**Fig. 2:**
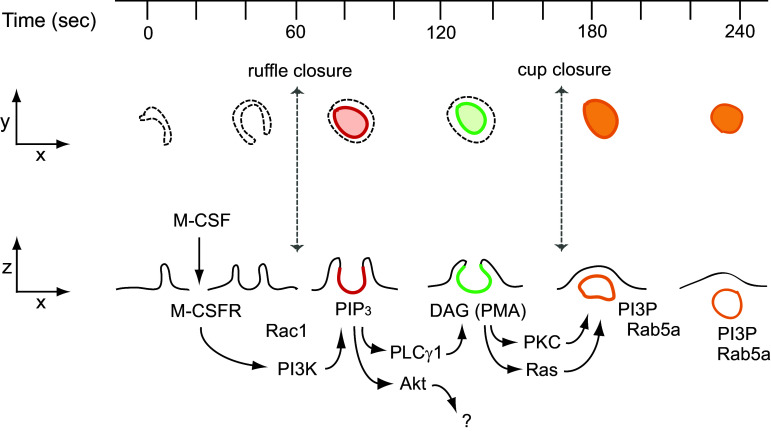
M-CSF-induced macropinocytosis. Interaction between M-CSF and the M-CSF receptor in macrophages activates Rac1 followed by induction of membrane ruffling. Some ruffles change into cup-like structures, in which activated PI3K then transiently generates PIP_3_ (red). PIP_3_ generation in the cup triggers the activation of PLCγ and Akt. Akt is not involved in macropinosome formation. PLCγ generates DAG in the cup (green), leading to activation of PKC and Ras. Both pathways contribute to cup closure, in which the macropinosome pinches off into the cytoplasm from the plasma membrane. Following cup closure, PI3P and Rab5a are localized at the macropinosomes (orange). Macropinosomes with these signals (orange) then move toward the center of the cells

Macropinocytosis was recognized long ago as a feature of growing cells [3, 85], but its essential role in growth was only established recently [7, 8, 40]. Many of the signaling molecules necessary for mTORC1 activation also contribute to macropinocytosis. The molecular mechanism of growth factor-induced macropinocytosis has been studied with a focus on the roles of small GTPases and phosphoinositides [1, 77, 86] (Fig. 2). Treatment of macrophages with their growth factor macrophage colony-stimulating factor (M-CSF) immediately induces irregular membrane ruffles at the cell margins which transform into “C”-shaped ruffles and then “O” shaped, cup-like structures. The open area at the top of the cup later closes to form a complete macropinosome [87]. The first stage of the closing process (C- to O-shaped ruffle) is termed ruffle closure, and the second phase (cup to macropinosome) is termed cup closure [1]. Fully closed macropinosomes move toward the center of the cell via the microtubule network and fuse with the lysosome [88] or, rarely, recycle to the plasma membrane [89]. Imaging of cells expressing fluorescent protein chimeric protein probes revealed a cascade of signals corresponding to the various stages of macropinosome formation. These temporally arranged signals were all restricted to the bowl of the macropinocytic cup, likely by structural barriers to lateral diffusion in the inner leaflet of the cup membrane [90]. Förster resonance energy transfer (FRET) microscopy showed that Rac1 was active within the cup domain immediately following ruffle closure [87]. Ratiometric fluorescence microscopy showed that cyan fluorescent protein (CFP)-labeled Rab5a was recruited to the cup membrane during cup closure and persisted on the macropinosome during its movement toward the lysosome [87]. Similarly, yellow fluorescent protein (YFP)-tagged Ras-binding domain of Raf (YFP-RBD), a probe to detect activated Ras [91], was recruited to macropinocytic cups in macrophages, suggesting that Ras is active during cup closure [92]. Similar macropinocytosis signaling patterns were also reported in other cell types following stimulation with platelet-derived growth factor (PDGF) [93–97]. Thus, as for activation of mTORC1, GTPases associated with membrane traffic are required for macropinocytosis.

Phosphoinositides are also essential for macropinocytosis. PI3K is required for all macropinocytosis except that stimulated by PMA [98, 99]. Fluorescence microscopy of macrophages stimulated with M-CSF showed transient recruitment of YFP-Btk-PH, which localizes PIP_3_, to the macropinocytic cup, indicating transient, localized PIP_3_ generation (PIP_3_ spike) [87, 92]. PI3K also regulates PDGF-induced macropinocytosis [100]. Live-cell imaging with fluorescent protein-tagged pleckstrin homology (PH)-domain chimeras demonstrated a signal transition from PI(4,5)P_2_ to PIP_3_ during epidermal growth factor (EGF)-induced macropinosome formation [86, 99]. Two well-known signal pathways are activated by PIP_3_: Akt and phospholipase C-γ (PLCγ). PLCγ is involved in macropinosome formation; Akt is not [101]. Imaging YFP-C1δ as a probe for the PLCγ product diacylglycerol (DAG) revealed transient generation of DAG in the cup [87, 101]. Live-cell imaging also showed that YFP-tagged protein kinase C (PKC)-α, which is activated by DAG, was recruited to cups [92]. The DAG mimetic PMA stimulates macropinocytosis in macrophages [102]. PMA-induced macropinocytosis is blocked by inhibitors of PKC and Ras but not by inhibitors of PLCγ or PI3K [101]. Additionally, the PIP_3_ spike was not observed in PMA-induced macropinocytic cups [40]. After cup closure, PI3P and Rab5a appeared on fully formed macropinosomes, which then moved toward the center of the cells [87]. The PKC inhibitor calphostin C blocked PDGF-induced macropinocytosis in MEFs [40]. Diacylglycerol kinase-ζ (DGKζ), which phosphorylates DAG to yield phosphatidic acid, is also necessary for macropinocytosis [103]. Knock-down of DGKζ attenuated PDGF-induced macropinocytosis. Therefore, DAG is a key signaling molecule involved in macropinocytosis. Together, these observations suggest that growth factor (GF)-induced macropinosome formation results from a signal cascade comprised of many molecules essential to growth control (Fig. 2).

The role of Ras in macropinosome formation remains undefined. Ras-induced pinocytosis was first described as a cellular response to injection of H-Ras [85]. H-Ras(G12V) expression induced membrane ruffles and macropinocytosis in HeLa cells, which could be inhibited by the actin polymerization inhibitor cytochalasin D or by co-expression of dominant-negative Arf6(T27N) [104]. K-Ras-induced macropinocytosis in fibroblasts was blocked by cytochalasin E or by the PI3K inhibitors wortmannin and LY294002 [5]. H-Ras-induced macropinocytosis in BHK-21 cells was blocked by wortmannin or by expression of dominant negative Rab5(S34N), but not by dominant negative Rac1(S17N) [105]. The differential association of K-Ras with PI3K p110 isoforms suggests roles for Ras in ruffling and macropinosome closure. However, MEFs deficient in K-Ras, H-Ras and N-Ras are capable of generating macropinosomes in response to PDGF [106], which suggests that macropinocytosis induced by oncogenic Ras may be an aberrant cellular behavior.

Phosphoinositide signals on macropinosomes were also observed during H-Ras(G12V)-induced macropinocytosis. Live-cell imaging using YFP-AktPH and YFP-PLCδ1-PH to localize PIP_3_ and PI(4,5)P_2_, respectively, showed that H-Ras(G12V)-induced macropinosomes in COS7 cells recruited both probe proteins and indicated that, like macropinocytosis in macrophages, PI(4,5)P_2_ was lost from macropinosomes before the PIP_3_ spike appeared [104]. Live-cell imaging showed co-localization of GFP-Akt and monomeric red fluorescent protein (mRFP)-H-Ras(G12V) at macropinosomes in COS7 cells [104]. Immunofluorescence staining showed that cells co-expressing H-Ras(G12V) and Arf6(Q67L) formed macropinosomes containing phosphorylated Akt [104]. YFP-Akt-PH was recruited to M-CSF-induced macropinocytic cups in macrophages [101] and to EGF-induced macropinocytic cups in A431 cells [99]. Moreover, GFP-Akt localizes to macropinosomes in LPS-stimulated macrophages [107]. Thus, Akt is activated at the macropinocytic cup and/or macropinosomes.

Ras is also required for macropinocytosis and cell growth in axenic strains of the free-living ameba *Dictyostelium discoideum* which are capable of growth in nutrient broth. Those strains exhibit Ras activity localized to macropinocytic cups, which are larger than cups in wild-type amebas due to a mutation in the Ras GAP neurofibromin [108, 109]. Thus, active Ras contributes to the morphogenesis of large macropinosomes necessary for nutrient acquisition and cell growth.

## Growth factor-induced macropinocytosis transfers amino acids into lysosomes to activate mTORC1

**Fig. 3:**
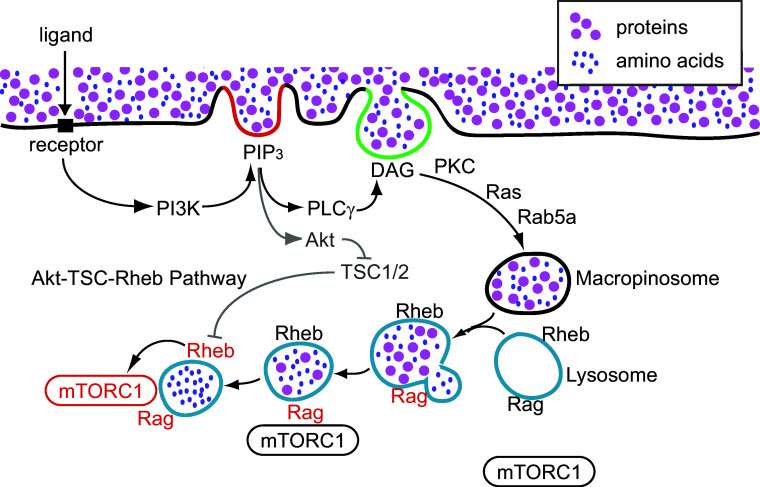
Macropinocytosis triggers mTORC1 activation. PI3K-generated PIP_3_ accumulates in macropinocytic cups (red line), activating Akt and PLCγ. PLCγ generates DAG in the cup (green line), leading to Ras- and PKC-dependent pathways that close the macropinosome. Extracellular nutrients internalized by the macropinosomes are delivered rapidly into lysosomes through fusion reactions. Nutrient transfer from macropinosomes to lysosomes induces Rag activation (black to red), followed by mTORC1 recruitment to lysosomes. Meanwhile, activated Akt inhibits TSC function in a cytosolic pathway independent of macropinocytosis, resulting in Rheb activation (black to red). Rheb directly activates mTORC1 on the lysosomal membranes (black to red)

Macropinocytosis rapidly and efficiently delivers extracellular solutes into lysosomes [110]. Given that growth factors induce both mTORC1 activation and macropinocytosis, and that they share many common GTPases and signaling molecules for their induction, we proposed a model in which macropinocytosis-mediated delivery of extracellular amino acids or protein to lysosomes is essential for mTORC1 activation (Fig. 3) [40]. Biochemical studies in murine macrophages showed that M-CSF treatment induced the PI3K–Akt–TSC–Rheb–mTORC1 pathway. Live-cell imaging and quantitative fluorescence microscopy showed that M-CSF-induced macropinocytosis delivered small extracellular molecules rapidly into lysosomes, where mTORC1 was recruited and activated. Inhibition of macropinocytosis by ethyl isopropylamiloride (EIPA) [111] or with the cytoskeleton inhibitors jasplakinolide and blebbistatin (J/B) blocked M-CSF-induced mTORC1 activation without inhibiting the PI3K–Akt pathway. These results suggest that macropinocytosis provides rapid amino acid trafficking into lysosomes to activate mTORC1. Like M-CSF-induced macropinocytosis, PMA-induced macropinocytosis also increased amino acid-dependent mTORC1 activation, but without inducing Akt phosphorylation. A role for macropinocytosis in mTORC1 activation was also demonstrated in MEFs. PDGF-induced mTORC1 activation by leucine (in the absence of glucose) was blocked by EIPA, J/B, or by knock-down of Rac1, in a manner independent of the Akt–TSC pathway. PDGF treatment increased mTOR recruitment to lysosomes, as determined by the co-localization of mTOR with LAMP2, a lysosomal membrane protein.

Based on these observations, it was proposed that growth factor stimulation induces macropinocytosis, leading to efficient uptake of essential amino acids via macropinosomes and subsequent delivery to the lysosome for mTORC1 activation (Fig. 3). Accordingly, growth factor- dependent mTORC1 activation is established by two distinct pathways: a PI3K–Akt–TSC–Rheb (cytosolic) pathway and a PI3K–macropinocytosis–Rag (vesicular) pathway. The cytosolic pathway is the classical Akt-dependent mTORC1 activation pathway described above: activated Akt induces TSC phosphorylation (TSC deactivation) and consequent activation of Rheb. In the vesicular pathway, PIP_3_ in macropinocytic cups localizes DAG synthesis and PKC activity, leading to macropinosome closure. Macropinosomes fuse with the tubular lysosomal network in macrophages or the lysosomes in MEFs, delivering ingested solutes such as proteins or amino acids. Amino acids transferred into the lysosome via macropinosome-lysosome fusion, or derived from hydrolysis of proteins in lysosomes, activate Ragulator and lead to subsequent activation of mTORC1 [40]. Therefore, growth factor receptor signaling organizes macropinosome formation, and the amino acids or proteins internalized by macropinocytosis signal to mTORC1 from inside lysosomes.

## The macropinosome as a signal platform for mTORC1 signaling

Macropinocytic cups and macropinosomes may also serve as structural platforms of signaling for cell growth. In addition to small GTPases, phosphoinositides are common signaling molecules involved in mTORC1 activation and macropinocytosis [76, 112]. Phosphoinositide kinase FYVE-type zinc finger containing (PIKFYVE) catalyzes the synthesis of PI(3,5)P_2_ from phosphatidylinositol 3-phosphate (PI3P) [113]. PI(3,5)P_2_ interacts with raptor [114], indicating its involvement in mTORC1 activation [112]. In 3T3-L1 adipocytes, depletion of PIKFYVE blocked insulin-induced activation of mTORC1 (as measured by S6K phosphorylation) without affecting Akt phosphorylation [114]. Myotubularin-related phosphatase 3 (MTMR3) dephosphorylates PI3P to phosphatidylinositol [115]. Depletion of MTMR3 in HEK293T cells increased nutrient-induced mTORC1 activation, suggesting that MTMR3 suppresses mTORC1 activity by depleting PI3P [116]. Therefore, the synthesis of PI3P or PI(3,5)P_2_ on macropinosomes could help recruit mTORC1 to the late endosome or lysosome.

The macropinocytic cup can also localize Akt phosphorylation. Like M-CSF, the chemokine CXCL12 induces both macropinocytosis and mTORC1 activation in macrophages [38]. Unlike the response to M-CSF, however, CXCL12-induced phosphorylation of Akt and S6K (a reporter of mTORC1 activity) was dependent on actin cytoskeleton rearrangement and the formation of macropinocytic cups. Live-cell imaging showed YFP-Akt-PH recruitment to the macropinocytic cup, and western blot analysis showed that the macropinocytosis inhibitors J/B and EIPA attenuated CXCL12-induced Akt phosphorylation. Thus, Akt phosphorylation in response to CXCL12 required the formation of a macropinocytic cup. Immunofluorescence microscopy showed that Akt was phosphorylated at membrane ruffles and macropinocytic cups. The PKCα/β-specific inhibitor Gö6976 blocked macropinocytosis and S6K phosphorylation without inhibiting membrane ruffling or cup formation, suggesting that PKCα and/or PKCβ are involved in cup closure. However, Gö6976 did not inhibit CXCL12-induced Akt phosphorylation. Together these studies indicated that CXCL12-induced macropinocytic cups are signal platforms for the Akt phosphorylation required for mTORC1 activation.

To what extent does the cytosolic pathway (Akt–TSC1/2–Rheb) require macropinocytosis? The sensitivity of Akt activation by CXCL12 to cytoskeleton-inhibitors differed from Akt activation in response to M-CSF or PDGF, which was not affected by such inhibitors. The organization of the macropinocytic cup may allow localized amplification of signals from some receptors, perhaps those that require multiple inputs for signal amplification. Circular ruffles create isolated domains of plasma membrane where signal propagation can occur [92], indicating the presence of barriers to lateral diffusion in the inner leaflet of the plasma membrane of cups [90]. Maximal Akt phosphorylation observed in response to CXCL12 was less than the level of Akt phosphorylation measured in response to M-CSF. Acute stimulation of cells with M-CSF (or PDGF) may generate sufficiently high concentrations of PIP_3_ that a spatially organized amplification is unnecessary. However, if receptors cannot generate high PIP_3_ concentrations, then phosphorylation of Akt may require a mechanism based on spatial confinement of signal amplification to macropinocytic cups. Consistent with this model, a recent study identified a role for Rac-dependent macropinocytosis in the activation of the PI3K subunit p110β by G-protein coupled receptors [117].

As described above, the TSC complex inhibits Rheb function at the lysosome [64, 73, 74]. When Akt and Erk phosphorylate TSC2, the TSC complex subsequently loses its GAP activity for Rheb [31, 32, 72]. This suggests that, within a few minutes of stimulation, signal components that phosphorylate Akt and Erk reach lysosomal structures and phosphorylate TSC2. In cells co-expressing H-Ras(G12V) and Arf6(Q67L), Erk is recruited to and phosphorylated at macropinosomes [104]. Erk localizes to late endosomes and lysosomes via the protein complex p18/p14/MP1 [118]. Since macropinosomes show late endosome characteristics at this stage, growth factor/chemokine-induced macropinosomes should recruit Erk via the p18/p14/MP1 protein complex during the maturation process. Given that another important function of the p18/p14/MP1 complex is to recruit mTORC1 to the lysosome as a Ragulator, we speculate that late stage macropinosomes recruit mTORC1 directly. Together, these reports indicate that macropinosomes deliver signaling molecules to the lysosome.

## How macropinocytosis could be essential to growth control

Macropinocytosis may be essential for the growth of metazoan cells [40]. Accordingly, when cells are growing in constant concentrations of growth factor, macropinosomes form stochastically as discrete units of growth factor signaling, and activation of mTORC1 follows after a bolus of extracellular protein or amino acids is delivered by macropinocytosis into the lysosomes. Moreover, Akt localization to cups and its continued association with fully formed macropinosomes could provide a route for Akt to reach its substrate tuberous sclerosis complex-1/2 (TSC1/2) on the lysosomal membrane. Thus, the magnitude of growth factor stimulation of mTORC1 may be determined in part by the volume of solute internalized by macropinocytosis, with feedback from a nutrient-sensing mechanism regulating the magnitude of Akt signaling on macropinosome membranes and the volume of nutrient delivered into the lysosome via macropinocytosis. This model predicts that macropinocytosis is necessary for cell growth and proliferation.

## Pathogenic functions of macropinocytosis in K-Ras-induced cancer

Dysregulation of Ras and mTORC1 are involved in cancer development [15, 29]. Pathologic functions of macropinocytosis in oncogenic K-Ras-expressing cancer cells have been described. Human carcinoma cells expressing K-Ras(G12C) or H-Ras(G12V) showed increased macropinocytosis, similar to NIH 3T3 cells expressing K-Ras(G12V). Extracellular proteins ingested by macropinocytosis in cells expressing oncogenic K-Ras were degraded and their constituent amino acids were used for anabolic metabolism [7]. The macropinocytosis inhibitor EIPA blocked albumin-dependent cell proliferation [7], indicating that ingestion of albumin by K-Ras(G12D)-induced macropinocytosis and subsequent hydrolysis of proteins in lysosomes were sufficient to provide the essential amino acids (EAA) necessary for cell proliferation [39]. Moreover, the growth of cells in nutrient-poor regions of pancreatic tumors was supported by scavenging of extracellular proteins [119]. Other groups have reported that H-Ras(G12V)-induced macropinocytosis is necessary for albumin-dependent cell growth of MEFs and that inhibition of mTORC1 activation increases the rate of macropinocytosis in carcinoma cells (MIA PaCa-2 K Ras mutant) [41, 42]. Additionally, inhibition of DOCK1, a Rac-activating protein required for macropinocytosis, reduces survival of Ras-driven cell growth [120]. Thus, macropinocytosis-mediated ingestion of extracellular protein is now considered a hallmark of cancer metabolism [121].

However, unlike the responses observed in macrophages and MEFs, mTORC1 activation by EAA in K-Ras transformed cells was not inhibited by EIPA [8]. This indicates that macropinocytosis in Ras-transformed cells is not the primary route by which free amino acids reach the cytosolic SESTRIN1/2 and CASTOR detection systems.

**Fig. 4:**
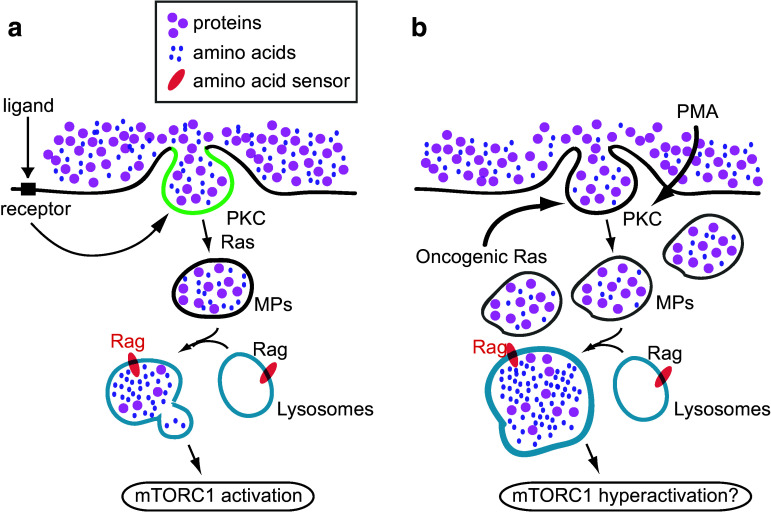
Two models of macropinocytosis-regulated mTORC1 activation. **a** Role of macropinocytosis in ligand-induced mTORC1 activation. Signals derived from DAG (green) modulate macropinosome (MP) formation via the activation of PKC and Ras. Formed macropinosomes convey extracellular nutrients into lysosomes, where Rag is activated. **b** Proposed hypothesis of the function of oncogenic protein-induced macropinocytosis and mTORC1 activation. Over-expression of oncogenic Ras continuously induces macropinosomes, resulting in an overload of nutrients in the lysosomes. Because of this, following Rag activation, mTORC1 is hyperactivated. PMA treatment directly induces PKC activation, which would also lead to increased nutrient uptake via macropinocytosis

In sum, these studies suggest that macropinosomes serve as organizational units of a signal transduction pathway that is induced by extracellular stimuli such as growth factors and chemokines (Fig. 4a). If this is the case, constitutive macropinocytosis induced by oncogenic K-Ras or cSrc may hyperactivate mTORC1, resulting in unrestrained growth (Fig. 4b). Similarly, the tumor promoting activity of PMA may be partly attributable to its activation of mTORC1 via macropinocytosis.

## Future directions

Significant questions remain to be answered about the relationship between macropinocytosis and mTORC1. To what extent does macropinocytosis support growth of non-neoplastic cells? Why is mTORC1 activation by EAA in K-Ras-transformed cells independent of macropinocytosis? Does membrane traffic unrelated to macropinocytosis regulate mTORC1 activity? Does the activity of mTORC1 or the nutrient status of the cell regulate macropinosome formation or fusion with the lysosomes? The studies of Palm et al. [8, 106] indicated that active mTORC1 inhibits protein delivery into lysosomes via macropinocytosis, whereas Nofal et al. [122], showed that mTORC1 activation does not affect degradation of extracellular protein. These studies suggest that mTORC1 or the cytosolic concentrations of amino acids regulate the uptake and degradation of extracellular solutes by macropinocytosis (i.e., heterophagy) in a manner analogous to its role in protein recycling and degradation by autophagy.

Alternative macropinocytosis-specific inhibitors are needed, both for better understanding of macropinocytosis biology and for the potential therapeutic manipulation of the macropinocytosis signaling pathway. Although EIPA does not block other types of endocytosis, such as phagocytosis and clathrin-dependent endocytosis, it is reasonable to expect it to affect other signal pathways related to cell growth and differentiation. Drugs targeting macropinocytosis could attenuate growth of neoplastic cells or related mosaic disorders resulting from mutations in the signals leading to mTORC1 [123].
